# Emergency Laparoscopic Repair of Giant Left Diaphragmatic Hernia following Minimally Invasive Esophagectomy: Description of a Case and Review of the Literature

**DOI:** 10.1155/2018/2961517

**Published:** 2018-09-12

**Authors:** Enrico Erdas, Gian Luigi Canu, Luca Gordini, Paolo Mura, Giulia Laconi, Giuseppe Pisano, Fabio Medas, Pietro Giorgio Calò

**Affiliations:** ^1^Department of Surgical Sciences, University of Cagliari, Cittadella Universitaria, SS554, Bivio Sestu, 09042 Monserrato, Italy; ^2^Department of Medical Sciences and Public Health, University of Cagliari, Cittadella Universitaria, SS554, Bivio Sestu, 09042 Monserrato, Italy

## Abstract

Postoperative diaphragmatic hernia (PDH) is an increasingly reported complication of esophageal cancer surgery. PDH occurs more frequently when minimally invasive techniques are employed, but very little is known about its pathogenesis. Currently, no consensus exists concerning preventive measures and its management. A 71-year-old man underwent minimally invasive esophagectomy for esophageal cancer. Three months later, he developed a giant PDH, which was repaired by direct suture via laparoscopic approach. A hypertensive pneumothorax occurred during surgery. This complication was managed by the anaesthesiologist through a high fraction of inspired O_2_ and several recruitment manoeuvres. The patient remained free of hernia recurrence until he died of neoplastic cachexia 5 months later. Laparoscopic repair of PDH may be safe and effective even in the acute setting and in the case of massive herniation. However, surgeons and anaesthesiologists should be aware of the risk of intraoperative pneumothorax and be prepared to treat it promptly.

## 1. Introduction

Postoperative diaphragmatic hernia (PDH) is a well-known complication of esophageal cancer surgery that can be associated with significant morbidity and mortality [1–6]. The risk of developing PDH after esophagectomy seems to be much higher when minimally invasive techniques are employed [2–5, 7]. Several mechanisms and risk factors may be involved in the pathogenesis of PDH, and many measures have been suggested in order to minimize its occurrence, although little evidence is available on this regard [1–11].

PDH may be detected incidentally during the oncological follow-up [1] or due to digestive, respiratory, and cardiac symptoms that can present acutely or chronically [2–5, 8].

The role of surgery in the treatment of asymptomatic PDH is a matter of debate, since the risk of developing symptoms or complications is poorly predictable [1–6, 8–10]. Surgery is clearly mandatory for symptomatic or complicated PDH, but currently, there is no consensus on the best method of repair [3–5, 7, 9]. The laparoscopic approach, with or without mesh, has recently gained a large consensus for the repair of primary hiatal hernias [3–5, 7, 9], but its use is still underreported regarding PDH [2–4].

The aim of this paper is to describe the technique, the advantages, and the pitfalls of the laparoscopic repair in a case of giant PDH with acute respiratory and cardiac symptoms presentation.

## 2. Case Report

A 71-year-old man underwent McKeown minimally invasive esophagectomy (MIE) for middle third esophageal cancer. The review of the operative report did not reveal any crus division or intentional hiatal widening. The operation lasted 5 hours and 20 minutes. The postoperative course was complicated by cervical esophagogastric anastomotic leak, dysphonia, and swallow dysfunction with subsequent aspiration pneumonia. The patient was successfully treated by long-term enteral feeding and intensive care and was discharged in stable condition on the 46^th^ postoperative day.

The histological examination revealed a stage IIIA (pT_2_N_2_M_0_) poorly differentiated squamous cell carcinoma.

**Figure 1:**
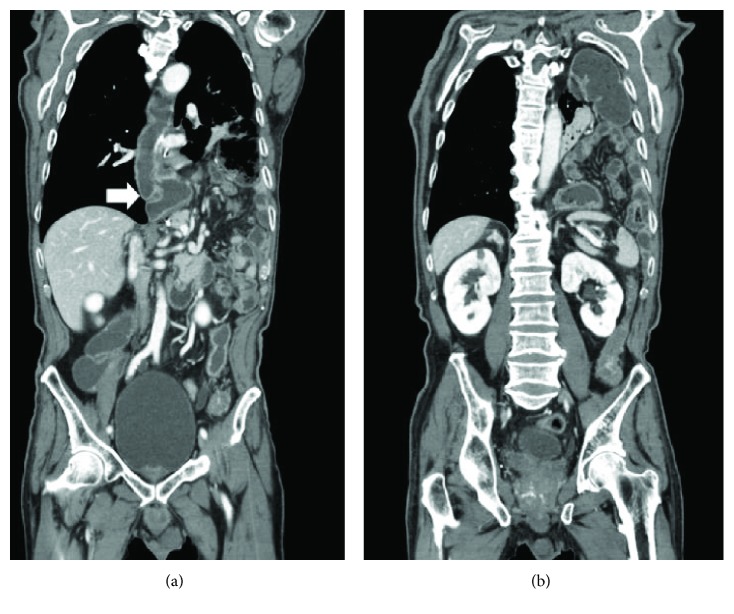
Coronal CT scan showing the massive transdiaphragmatic herniation of abdominal viscera in the left hemithorax. (a) The gastric conduit on the right of the herniated viscera (arrow). (b) The left hemithorax totally occupied by the abdominal viscera.

**Figure 2:**
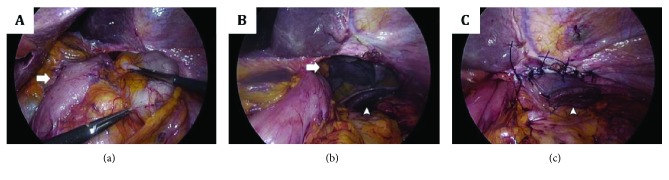
Intraoperative view of the diaphragmatic hernia. (a) The gastric conduit is visible to the right of the herniated viscera (arrow); (b) after the reduction of the hernia content, the diaphragmatic defect, the left lung (arrow), and the spleen (arrowhead) are clearly visible; (c) the diaphragmatic defect repaired by direct suture.

At 3-month follow-up, multiple recurrences to right paratracheal lymph node, anterior chest wall, and right adrenal gland had been detected at positron emission tomography (PET) and computed tomography (CT) scan, and thus the patient was referred to the Oncological Unit to start adjuvant chemotherapy. However, a few days after admission, he complained of acute onset of severe upper abdominal pain, nausea, and dyspnea, which occurred immediately after a prolonged effort at defecation. On clinical examination, he was pale, bradycardic (35 beats per minute), hypotensive (blood pressure: 60/40 mmHg), and tachypneic (26 breaths per minute). The abdomen appeared excavated with diffuse tenderness and impaired bowel sound. Vesicular breath sounds were considerably reduced over the entire left hemithorax. After achieving satisfactory haemodynamic stability with high flow oxygen and iv fluid therapy, a CT scan with contrast was performed which documented the near-complete herniation of the small bowel, transverse colon, and greater omentum through a large defect (8.5 × 5 cm) of the left hemidiaphragm, resulting in ipsilateral massive lung collapse (Figure 1). The patient was immediately transferred to our surgical unit to undergo emergency relaparoscopy for a giant diaphragmatic hernia. Due to the coexistence of several medical illnesses (alcoholic liver disease, chronic renal failure, and arterial hypertension), the patient was considered at high anesthesiological risk (class III, according to the American Society of Anaesthesiology Physical Status Classification System). After general anaesthesia, a double-lumen endotracheal tube was inserted in order to selectively ventilate the right lung in case of conversion to open surgery. The Hasson technique was used to create pneumoperitoneum 2 cm above the umbilicus, and three operative trocars were placed in the same sites of the previous operation (one 12 mm trocar in the left hypochondrium and two 5 mm trocars, respectively, under the xiphoid and in the right hypochondrium). After CO_2_ insufflation to a pressure of 12 mmHg, the abdominal cavity was explored with a 30° laparoscope. The entire hiatal anatomy was initially hidden by the herniated small bowel and transverse colon, which were gradually reduced into the abdominal cavity with progressive gentle traction (Figure 2(a)). This allowed for the identification of a large defect of the left hemidiaphragm, with its long axis oriented transversely from the gastric conduit to the superior border of the spleen. The left diaphragmatic pillar was not recognizable, and there was no evidence of hernia sac and diaphragmatic pleura (Figure 2(b)). Due to the direct communication between the peritoneal cavity and left pleural space, a hypertensive left pneumothorax was gradually developed, which mandated high fractions of inspired O_2_ and several vital capacity recruitment manoeuvres until the repair had been completed. The hernia orifice was primarily closed by approximating its anterior and posterior borders through a series of interrupted nonabsorbable stitches (0-Ethibond ™, Ethicon, Somerville, NJ, USA) (Figure 2(c)). In this way, a neo-hiatus was created, paying attention not to overtight the gastric conduit and preserve its vascular supply. At the end of the procedure, no drain tube was placed in the left chest, because the hemodynamic instability and the hypoxemia were definitively resolved. Before extubation, a chest X-ray showed a completely reexpanded left lung.

The postoperative course was uneventful, and the patient was discharged 7 days after surgery. No signs of hernia recurrence were identified on CT scan at 3-month follow up. The patient died 2 months later due to neoplastic cachexia.

## 3. Discussion

PDH is one of the most dangerous complications of esophageal cancer surgery [2–5, 10]. The incidence of PDH varies widely from 0% to 26% depending on the type of operation, stage of esophageal cancer, duration and modality of follow-up, and whether or not asymptomatic patients were considered [2–7, 10].

**Figure 3:**
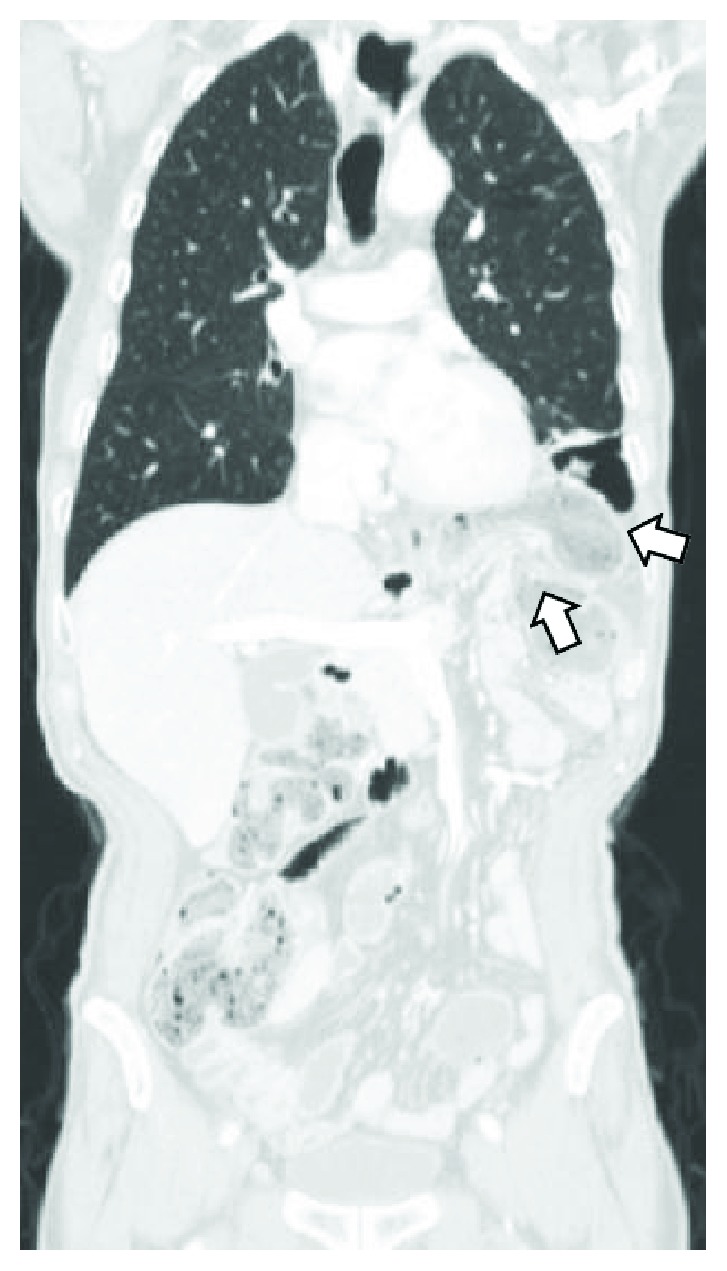
CT scan performed during the oncological follow-up. The diaphragmatic hernia was clearly visible but has not been described in the radiological report. Left side diaphragmatic defect (lower arrow); abdominal viscera in the left hemithorax (upper arrow).

Until a decade ago, PDH was rarely reported, but since the introduction and progressive uptake of minimally invasive esophagectomy (MIE), the range of incidence rose from 0.2–6% to 2.2–26% [2–5, 7, 10]. However, whether MIE should be considered in itself, a risk factor for PDH occurrence is difficult to establish. The hiatal enlargement during esophagectomy is probably the main predisposing factor and may occur in both open and laparoscopic approach. Indeed, the partial resection of esophageal hiatus may be necessary to comply with oncological principles, and crura may need to be divided to allow the passage of the gastric conduit and avoid compression to its vascular supply [1, 5, 10]. Furthermore, hiatal widening can also result from the stretching of the crus muscles following transhiatal manoeuvres during both open and laparoscopic approach. A recent meta-analysis showed that the incidence of PDH after open esophagectomy range from 0% to 10% but rise up to 20% when the transhiatal technique is employed [2]. Finally, the increased incidence of PDH may also be due to the improved survival of patients submitted to neoadjuvant oncological therapies [5, 9]. However, MIE carries at least two other potential risk factors compared to an open approach. The first is less amount of postoperative peritoneal adhesions that can favour the passage of viscera also through a small hiatal orifice due to the suction effect exerted by the chest during respiration [1, 4, 5, 7, 10]. The second is the long-lasting pneumoperitoneum, which may result in a severe stretching of crus muscles with consequent hiatal enlargement [2, 10]. Our findings support these theories, since no intentional hiatal enlargement or crus division was performed during MIE, but pneumoperitoneum lasted several hours. Furthermore, during the second operation, no significant visceral adhesions were found. The lack of peritoneum and pleura at the hiatal orifice suggests that the hernia occurred immediately after surgery and remained unnoticed until the occurrence of acute clinical manifestations, which is about 3 months later. The reviews of CT scan performed during the oncological follow-up confirm that PDH was already present before its clinical onset but was not reported by the radiologist (Figure 3). On this regard, Ganeshan et al. [6] demonstrated that this condition is strongly underreported, with only 10% of the cases detected by the radiologists in their first CT study. The authors argue that, during the oncological follow-up, radiologists' attention is focused on the detection of cancer recurrences, and, as a consequence, a small asymptomatic PDH can go unnoticed or underestimated. However, the benefit of detecting and treating small asymptomatic PDH is not fully proven. The danger that hernia enlarges over time, with the risk of incarceration or strangulation, would suggest surgical treatment of all PDH at the time of the diagnosis, with the only exception of patients with significant comorbidity or short life expectancy. On the other hand, the risk of possible acute complications, which is not currently quantifiable, must be weighed against the high operative morbidity and mortality rate, which account up to 60% and 14%, respectively [2–5, 10]. However, the poor outcome of PDH repair is most likely related to the high rate of emergency surgery (20–60% of cases) and to the fact that, still today, many operations are performed via an open approach [2–5]. Matthews et al. [5] reported an overall postoperative mortality of 13% after PDH repair, but it was 20% in patients who required emergency surgery and 0% in those operated on an elective basis. More generally, it has been definitely proven that laparoscopic repair of hiatal hernia in the elective setting is associated with a rate of perioperative morbidity and mortality much lower than reported for the open approach [12]. For these reasons, there is a growing consensus that even asymptomatic PDH should be repaired, unless patients are unfit for surgery or have a short life expectancy [4, 8, 10].

As in the present case, the laparoscopic repair of PDH has proved to be safe and effective even in the acute setting [3, 4, 7, 9]. Laparoscopy has several advantages over the conventional open repair, such as reduced postoperative pain and enhanced recovery time. Furthermore, it allows to rule out any cancer recurrences prior to consider whether and how to perform hernia repair and to better visualise and preserve the herniated contents and the vascular supply of the gastric conduit [2, 4, 9]. Unfortunately, a high rate of conversion to open surgery has been reported (up to 42%) for various reasons, including bowel gangrene, splenic injury, inability to reduce the herniated contents, and hypertensive pneumothorax [2, 5, 11]. In the presented case, hypertensive pneumothorax occurred due to the lack of hernia sac and diaphragmatic pleura. However, the provision of high oxygen fractions and the application of several recruitment manoeuvres allowed the procedure to be completed laparoscopically. As reported by Fumagalli et al. [11], the insertion of an intercostal drain may be used to treat intraoperative pneumothorax, but it may cause loss of pneumoperitoneum, creating suboptimal conditions for the continuation of surgery [13]. As described by Joris et al. [14], positive end-expiratory pressure may be used as an effective alternative to chest tube placement, allowing the correction of the respiratory changes associated with pneumothorax.

Another controversial issue is related to the method of hernia repair. Currently, there is moderate evidence that mesh cruroplasty is associated with a lower risk for short-term recurrence as compared to direct suture, but these data have not been confirmed in the long-term [15, 16]. On the other hand, the use of mesh may result in severe complications such as visceral erosion, pericardial tamponade, and mesh infection [7, 15]. For these reasons, direct suture remains the standard method of hiatal repair for most surgeons, while mesh cruroplasty is confined to large hiatal defect for which a tension-free closure cannot be achieved [1, 4–7]. In the present case, the decision to perform a primary repair was based not only on technical reasons but also on the short life expectancy of the patient.

Up to now, several techniques have been suggested in order to reduce the risk of PDH, but little data are available on their effectiveness. Minimising the hiatal widening and repairing preexisting or iatrogenic large hiatal defect are probably the mainstays of the prevention [2, 3, 5, 6, 10, 11]. Some authors advocate the use of a biological mesh to close a residual large hiatal defect [3, 17]. However, there is no evidence on the effectiveness of this strategy. Other accessory measures may be represented by crural fixation of the gastric conduit and anterior abdominal wall colopexy [1, 5, 11].

In conclusion, PDH is a serious complication of esophageal cancer surgery that seems to occur more frequently after MIE. Early diagnosis is difficult to establish, since clinical manifestation may be lacking and small PDH may be overlooked at CT scan during the oncological follow-up. Elective repair is advisable even for asymptomatic patients in order to prevent severe complications such as intestinal obstruction, gastric ischemia, and acute respiratory distress. Laparoscopic repair, with or without prosthetic reinforcement, may be safe and effective even in the acute setting and in the case of massive intrathoracic herniation. However, surgeons and anaesthesiologists should be aware of the risk of intraoperative pneumothorax and be prepared to recognise and treat it promptly.
